# lncRNA GAS5 enhances G1 cell cycle arrest via binding to YBX1 to regulate p21 expression in stomach cancer

**DOI:** 10.1038/srep10159

**Published:** 2015-05-11

**Authors:** Yongchao Liu, Jing Zhao, Wenhong Zhang, Jun Gan, Chengen Hu, Guangjian Huang, Ying Zhang

**Affiliations:** 1 Department of General Surgery, Huashan Hospital, Fudan University, Shanghai, China; 2 Department of Infectious Diseases, Huashan Hospital, Fudan University, Shanghai, China; 3 Department of Molecular Microbiology and Immunology, Bloomberg School of Public Health, Johns Hopkins University, Baltimore, Maryland, USA

## Abstract

Long non-coding RNAs (lncRNAs), which have evolved as important gene expression modulators, are involved in human malignancies. The down-regulation of lncRNA growth arrest specific transcript 5 (GAS5) has been reported in several cancers, however, the underlying mechanism of lncRNA GAS5 in stomach cancer is poorly understood. In this study, we found that lncRNA GAS5 had lower expression in stomach cancer tissues than the normal counterparts. lncRNA GAS5 was shown to interact with Y-box binding protein 1 (YBX1), and lncRNA GAS5 knockdown was shown to accelerate YBX1 protein turnover without affecting YBX1 transcription. lncRNA GAS5 down-regulation reduced the YBX1 protein level, which decreased YBX1-transactivated p21 expression and abolished G1 phase cell cycle arrest in stomach cancer. These results delineate a novel mechanism of lncRNA GAS5 in suppressing stomach carcinogenesis, and the lncRNA GAS5/YBX1/p21 pathway we discovered may provide useful targets for developing lncRNA-based therapies for stomach cancer.

Stomach cancer is one of the most aggressive malignancies and represents the second leading cause of cancer death worldwide^1^,^2^. Much progress has been made in the molecular understanding of stomach cancer, including tumor suppressor gene mutations, aberrant protein expression and cancer stem cell identification^3^. However, effective strategies to decrease the incidence and mortality of stomach cancer remain lacking^4^. Thus, understanding additional carcinogenesis mechanism of stomach cancer is urgently needed for developing new therapies for clinical application^4^,^5^.

Currently, large-scale genome-wide projects have found that nearly 2/3 of mammalian genomic DNA is transcribed, however, less than 2% is translated into proteins^6^. These non-coding RNAs function as regulators of gene expression, this regulation was previously thought to be the role of proteins. This finding indicated a new RNA-based gene regulation mechanism that complements the central dogma^7^. In addition to various small non-coding RNAs such as microRNAs and siRNAs, a great proportion of the transcriptome generates RNA transcripts with lengths that are more than 200 nucleotides. These RNA molecules are defined as lncRNAs, which have as yet unknown RNA-based regulatory mechanisms^8^,^9^. lncRNAs are able to interact with various biomolecules, including DNA, RNA and proteins, to regulate gene expression at transcriptional, post-transcriptional and epigenetic levels^8^,^10^. lncRNAs are involved in many human diseases^11^,^12^, particularly in the development and progression of cancers. For instance, the oncogene c-Myc can induce lncRNA H19, which decreases the expression of the tumor suppressor microRNA let-7a, thus promoting carcinogenesis^13^,^14^,^15^. Additionally, lncRNA HOTAIR overexpression indicates poor prognoses in various human malignancies through multiple mechanisms^16^,^17^. Therefore, pivotal roles of lncRNAs in gene regulation have been acknowledged in recent studies^7^.

Among all the known cancer-related lncRNAs, lncRNA GAS5 is a growth suppressor^18^ that is up-regulated when cell growth inhibition is caused by starvation or rapamycin in T cells^19^,^20^. Recently, the exon 12-derived stem-loop region of lncRNA GAS5 was found to mimic the glucocorticoid receptor response element (GRE) structurally, and lncRNA GAS5 was shown to compete with GRE to associate with the DNA-binding domain of glucocorticoid receptor (GR). Additionally, lncRNA GAS5 acts as decoy of GRE to inhibit the downstream gene expression of GRE such as cIAP2 and triggers apoptosis during starvation^21^. Moreover, in bladder cancer cells and pancreatic cancer cells, lncRNA GAS5 functions as a tumor suppressor by regulating the expression of the oncogene cyclin-dependent kinase (CDK) 6^22^. However, few reports have examined the role of lncRNA GAS5 in stomach cancer. Therefore, we performed this study to explore the function and regulatory mechanism of lncRNA GAS5 in stomach cancer. Our results revealed that lncRNA GAS5 expression was down-regulated in stomach cancer tissues compared with that in paired normal counterparts. lncRNA GAS5 could interact with the transcriptional activator YBX1, the down-regulation of lncRNA GAS5 expression accelerated YBX1 protein turnover, which subsequently decreased the expression of the cell cycle regulator p21 and abolished cell cycle arrest at the G1 phase. These results provide new insight regarding the mechanism of lncRNA GAS5 suppressing stomach cancer pathogenesis and have implications for the development of lncRNA-based cancer treatments.

## Results

### lncRNA GAS5 is accumulated in response to starvation and rapamycin stimulation but is down-regulated in stomach cancer tissues

**Figure 1:**
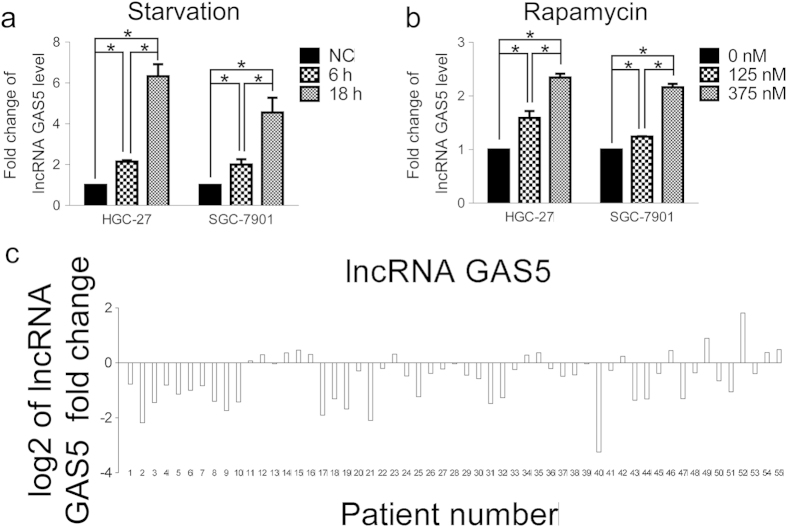
lncRNA GAS5 is accumulated in response to starvation and rapamycin stimulation, but is down-regulated in stomach cancer tissues. (**a**) In HGC-27 and SGC-7901, the expression of lncRNA GAS5 was increased after starvation without FBS in the 1640 cell culture medium. Cell without starvation treatment was NC (negtive control). (**b**) In HGC-27 and SGC-7901, the expression of lncRNA GAS5 was increased after rapamycin stimulation. (**c**) The log2 value of the lncRNA GAS5 relative fold change in 55 paired stomach cancer specimens. The relative fold change was calculated by 2^−ΔΔCt^.*, p < 0.05.

Previous studies have found that the lncRNA GAS5 expression level increases when T cell growth is suppressed by starvation or rapamycin^19^,^20^. In two stomach cancer cell lines, i.e., HGC-27 and SGC-7901, we also found that lncRNA GAS5 was up-regulated in an obvious time-dependent manner when starved for 6 h and 18 h (Fig. 1a). Furthermore, after treatment with 125 nM and 375 nM rapamycin, the expression level of lncRNA GAS5 significantly increased in a concentration-dependent manner (Fig. 1b). These results suggest that lncRNA GAS5 participates in cell growth inhibition in stomach cancer. Then, we detected lncRNA GAS5 expression in 55 paired stomach cancer and normal adjacent tissue specimens. As shown in Fig. 1c and Supplementary Table S1 online, lncRNA GAS5 expression was significantly down-regulated in 74.5% of cancer tissues compared with paired normal counterparts (p < 0.05). These results indicate that lncRNA GAS5 is a potential tumor suppressor in stomach cancer and that decreased lncRNA GAS5 expression may be associated with abnormal cell growth during stomach cancer development.

### lncRNA GAS5 knock-down abolishes cell cycle arrest at the G1 phase

**Figure 2:**
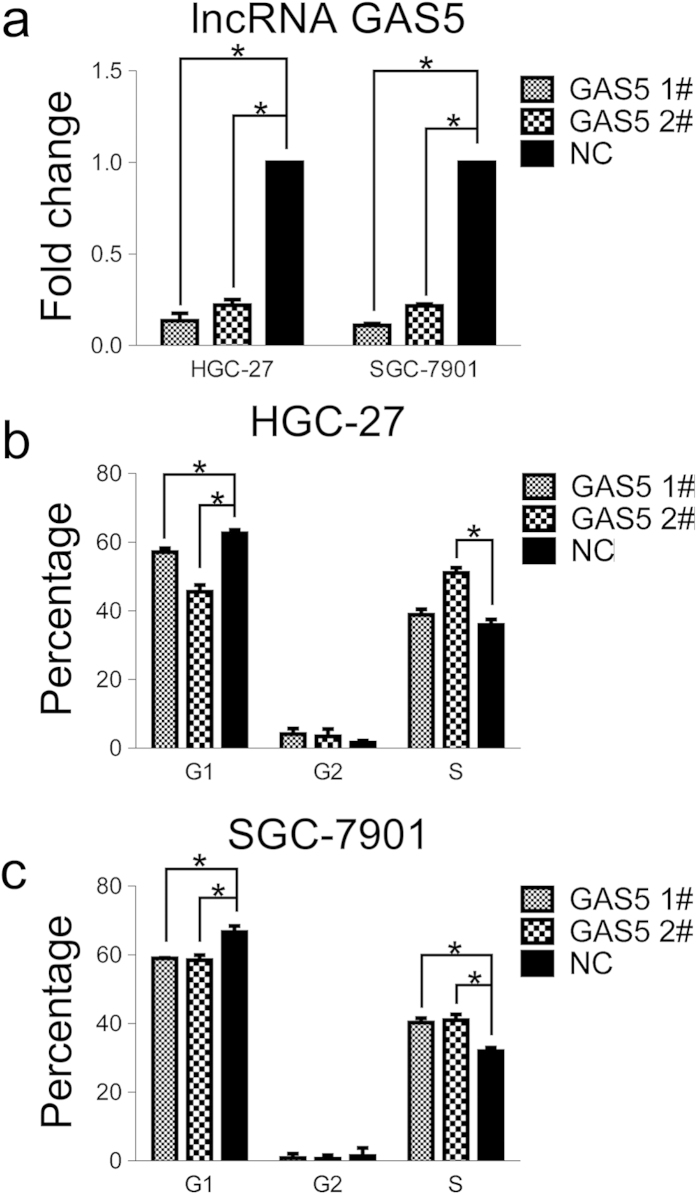
lncRNA GAS5 knock-down abolishes cell cycle arrest at G1 phase. (**a**) The lncRNA GAS5 was down-regulated in both cell lines after treatment with two siRNAs against lncRNA GAS5. (**b**) and (**c**) The percentage of cells arresting in G1 phase was decreased in HGC-27(b) and in SGC-7901(c). NC was the negative control sequence for siRNAs.*, p < 0.05.

To study the effect of lncRNA GAS5 down-regulation on cellular function, two siRNA oligos against lncRNA GAS5 were employed in HGC-27 and SGC-7901 cells. Upon siRNA transfection, lncRNA GAS5 was significantly depleted in both cell lines (Fig. 2a). Additionally, the percentage of cells in G1 phase significantly decreased with lncRNA GAS5 knockdown in both cell lines (Fig. 2b,c). These results indicate that lncRNA GAS5 down-regulation can abolish cell cycle arrest at the G1 phase.

### lncRNA GAS5 interacts with the transcriptional activator YBX1

**Figure 3:**
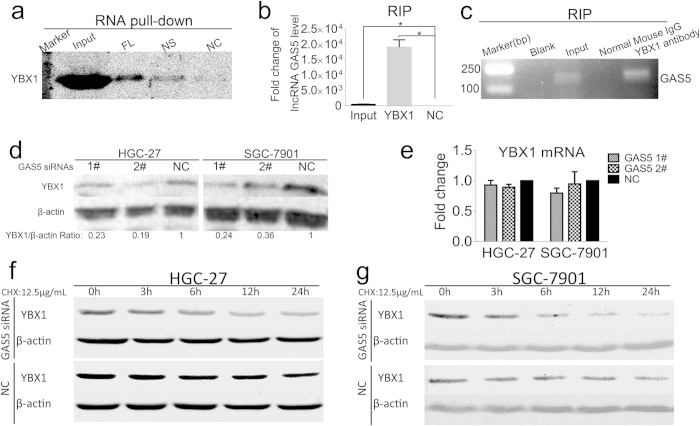
lncRNA GAS5 interacts with the transcriptional activator YBX1. (**a**) Western blot detected the YBX1 in the GAS5 pull-down complex. GAS5-FL was the biotin labeled lncRNA GAS5, NS was the biotin labeled non-sense RNA with similar length to GAS5, NC was the lncRNA GAS5 without biotin label. (**b**) qRT-PCR confirmed that lncRNA GAS5 was accumulated in YBX1-precipatated protein sample. (**c**) The agarose gel electrophoresis graph showed PCR products of RIP. (**d**) YBX1 protein level was decreased with lncRNA GAS5 knock-down. (**e**) The YBX1 mRNA levels were not affected by lncRNA GAS5 knock-down. NC was the negative control sequence for siRNAs. (**f**) and (**g**) lncRNA GAS5 had effect on YBX1 protein turnover under the treatment of CHX at the indicate interval in HGC-27 cells (**f**) and in SGC-7901 cells (**g**). *, p < 0.05.

To explore the mechanism by which lncRNA GAS5 regulates the cell cycle, RNA pull-down assay was performed with the HGC-27 cell extracts to identify whether some proteins are involved in this process. The specific bands of potential lncRNA GAS5-bound proteins were compared with NS and NC and determined by mass spectrometry (MS), as shown in Supplementary Figure S1 online. NS was the biotin labeled non-sense RNA with similar length to GAS5, and NC was the GAS5 RNA without biotin label. Among these MS-identified proteins, YBX1 had the highest frequency and score (see Supplementary Table S2 online), which indicated that lncRNA GAS5 might bind to the transcriptional activator YBX1. To confirm the MS result, the GAS5 pull-down complex was detected by Western blot analysis using YBX1 antibody, and a stronger YBX1 signal could be observed with the GAS5 pull-down complex than with NS and NC, as shown in Fig. 3a. YBX1 is a DNA/RNA binding protein, which may account for the weak YBX1 signal observed in the NS lane (Fig. 3a). To verify the interaction between lncRNA GAS5 and YBX1 further, we performed RNA immunoprecipitation (RIP) assay. As shown in Fig. 3b,c, the YBX1 protein could specifically interact with lncRNA GAS5. To clarify whether the association between lncRNA GAS5 and YBX1 affected YBX1 expression, both YBX1 mRNA and protein levels were detected when lncRNA GAS5 was knocked down. We found that lncRNA GAS5 depletion reduced the YBX1 protein level (Fig. 3d) without affecting its mRNA level (Fig. 3e). Next, we determined whether lncRNA GAS5 expression affected YBX1 protein turnover. Cycloheximide (CHX) was used to block *de novo* protein synthesis, and the YBX1 protein level was examined at the indicated intervals via Western blot analysis. As shown in Fig. 3f,g, lncRNA GAS5 depletion accelerated YBX1 protein turnover in both HGC-27 and SGC-7901 cells. These findings suggest that lncRNA GAS5 can interact with the transcriptional activator YBX1 and that the down-regulation of lncRNA GAS5 may accelerate YBX1 degradation.

### YBX1 depletion reduces G1 phase arrest by decreasing p21 expression

**Figure 4:**
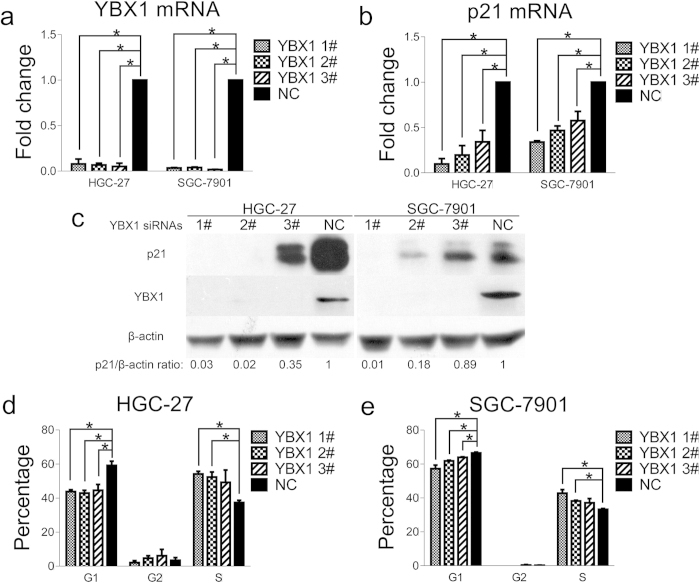
YBX1 depletion reduces G1 phase arrest by decreasing p21 expression. (**a**) The YBX1 mRNA level was down-regulated in both cell lines after treatment with three siRNAs against YBX1. (**b**) The p21 mRNA level was down-regulated with YBX1 silence in both cell lines. (**c**) The protein levels of YBX1 and p21 were both down-regulated in HGC-27 and SGC-7901 cell lines upon transfection with three siRNAs against YBX1. (**d**) and (**e**) The percentage of cells arresting in G1 phase was decreased in HGC-27 (**d**) and in SGC-7901 (**e**). NC was the negative control sequence for siRNAs. *, p < 0.05.

p21 is a well-known G1 phase regulator, and YBX1 transactivates p21 expression in prostate cancer^24^. To assess whether YBX1 could affect p21 and the cell cycle in stomach cancer, three YBX1 siRNA oligos were transfected into HGC-27 and SGC–7901 cells. As a result, p21 mRNA and protein levels were significantly down-regulated upon YBX1 knockdown (Fig. 4a–c). Moreover, the percentage of cells in G1 phase significantly decreased in both cell lines upon YBX1 depletion (Fig. 4d,e). These findings suggest that the down-regulation of lncRNA GAS5 expression in stomach cancer may reduce the YBX1 protein level by accelerating YBX1 protein turnover and that the low YBX1 abundance can decrease p21 expression, thus abolishing the G1 phase arrest.

### p21 expression correlates with lncRNA GAS5 expression in stomach cancer tissues

**Figure 5:**
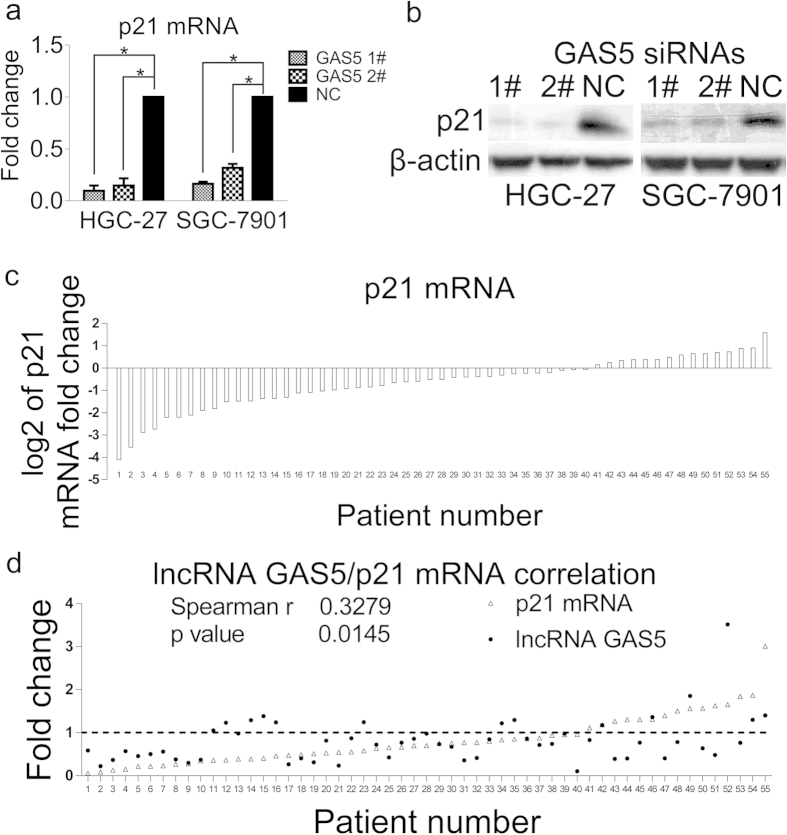
p21 expression correlates with lncRNA GAS5 expression in stomach cancer tissues. (**a**) The p21 mRNA levesl were down-regulated in both cell lines upon transfection with two siRNAs against lncRNA GAS5. (**b**) The protein levels of p21 were decreased with lncRNA GAS5 knock-down. (**c**) The log2 value of the p21 mRNA relative fold change in 55 paired stomach cancer specimens. (**d**) The relative fold change and correlation of lncRNA GAS5 and p21 mRNA levels in 55 stomach cancer specimens by Spearman correlation test. The relative fold change was calculated by 2^−ΔΔCt^. NC was the negative control sequence for siRNAs. *, p < 0.05.

Then, we found that the p21 mRNA and protein levels significantly decreased in HGC-27 and SGC-7901 cell lines after these cell lines were transfected with two lncRNA GAS5 siRNAs (Fig. 5a,b). In 55 paired stomach cancer and normal adjacent specimens, the p21 mRNA level was significantly down-regulated in 72.7% of cancer tissues compared with paired normal tissues (Fig. 5c and Supplementary Table S1 online), which was positively correlated with decreased lncRNA GAS5 expression in tissue specimens (Fig. 5d). These results suggest that lncRNA GAS5-induced cell cycle arrest may be mediated by p21 and that p21 may act as a downstream regulator in the lncRNA GAS5/YBX1 pathway.

### YBX1 plays a critical role in lncRNA GAS5-mediated p21 regulation to cause G1 phase cell cycle arrest

**Figure 6:**
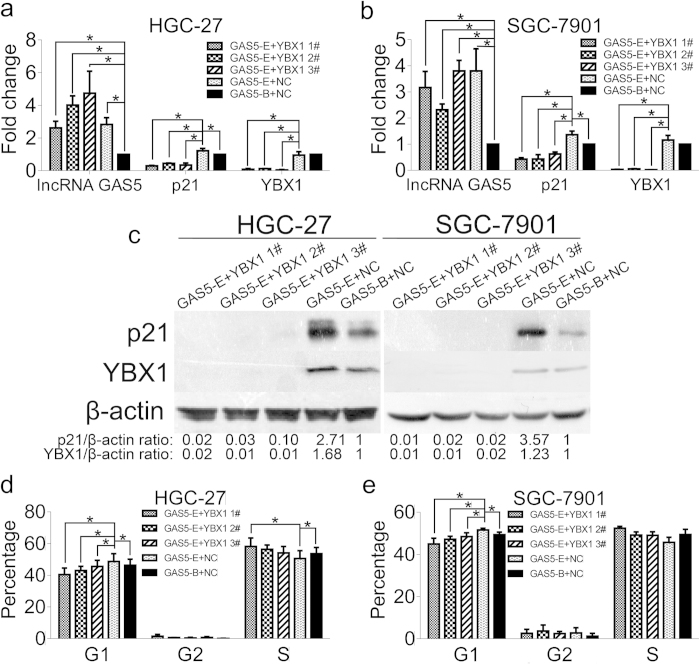
YBX1 plays a critical role in lncRNA GAS5-mediated p21 regulation to cause G1 phase cell cycle arrest. (**a**) and (**b**) The mRNA levels of lncRNA GAS5, p21 and YBX1 after cotransfection in HGC-27 cell line (**a**), and in SGC-7901 cell line (**b**). (**c**) The protein levesl of p21, YBX1 and β-actin of HGC-27 and SGC-7901 after the indicated cotransfection treatments. (**d**) and (**e**) The cell cycle alteration after cotransfection as indicated in HGC-27 (**d**) and in SGC-7901 (**e**). GAS5-E was lncRNA GAS5 expression plasmid, and GAS5-B was lncRNA GAS5 blank empty plasmid as control. YBX1 1#, YBX1 2# and YBX1 3# were three siRNA oligos to knock down YBX1. NC was the siRNA negative control. The details of cotranfection were as follows: Group1: GAS5-E + YBX1 1#; Group2:GAS5-E + YBX1 2#; Group3:GAS5-E + YBX1 3#; Group4:GAS5-E + NC; Group5:GAS5-B + NC. *, p < 0.05.

To confirm the mechanism by which the lncRNA GAS5/YBX1/p21 pathway regulates the cell cycle further, HGC-27 and SGC-7901 cells were cotransfected with YBX1 siRNAs and lncRNA GAS5 plasmid as indicated in Fig. 6. The lncRNA GAS5 expression plasmid was termed as GAS5-E and lncRNA GAS5 blank empty plasmid was named GAS5-B. When lncRNA GAS5 was overexpressed, the YBX1 mRNA level was not affected, however, the YBX1 protein level increased (Fig. 6a,c, group 4<GAS5-E + NC> vs group 5<GAS5-B + NC>). Simultaneously, the p21 mRNA and protein levels increased significantly (Fig. 6a,c, group 4<GAS5-E + NC> vs group 5<GAS5-B + NC>). However, cotransfection with YBX1 siRNAs and lncRNA GAS5 plasmid reversed the lncRNA GAS5-mediated increase in p21 mRNA and protein levels (Fig. 6a,c, groups 1-3<GAS5-E + YBX1 #1/#2/#3> vs group 4<GAS5-E + NC>). lncRNA GAS5 overexpression enhanced G1 phase arrest (Fig. 6d,e, group 4<GAS5-E + NC> vs group 5<GAS5-B + NC>), but cotransfection with YBX1 siRNAs and lncRNA GAS5 plasmid reversed these effects (Fig. 6d,e, groups 1-3<GAS5-E + YBX1 #1/#2/#3> vs group 4<GAS5-E + NC>). The percentage of cells that arrested in G1 phase was consistent with p21 expression (Fig. 6). These findings suggest that YBX1 plays a critical role in the lncRNA GAS5-mediated regulation of p21, which causes G1 phase cell cycle arrest, and that a novel cell cycle regulation mechanism involving the lncRNA GAS5/YBX1/p21 pathway exists in stomach cancer pathogenesis.

### lncRNA GAS5 mutant fails to arrest cell cycle at the G1 phase

**Figure 7:**
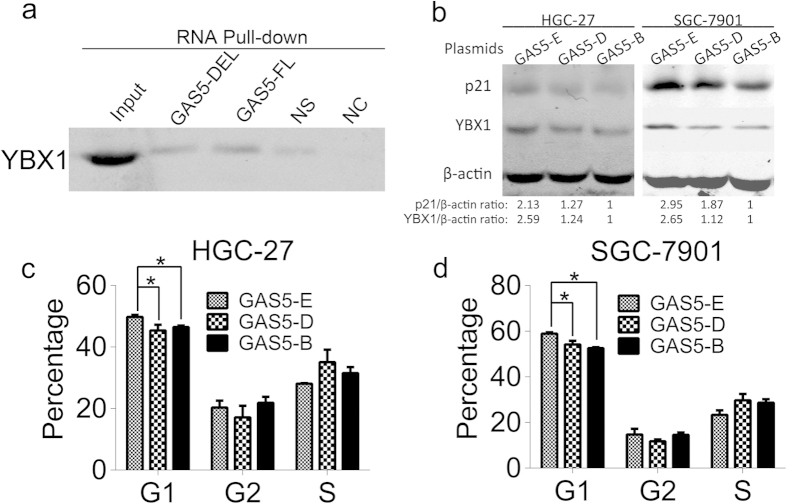
LncRNA GAS5 mutant fails to arrest cell cycle at the G1 phase. (**a**) Western blot detected YBX1 from GAS5-DEL and GAS5-FL RNA pull-down complexes. GAS5-DEL was biotin labeled lncRNA GAS5 mutant with its exon 12 deletion. GAS5-FL was biotin labeled full length lncRNA GAS5. NS was the biotin labeled non-sense RNA with similar length to GAS5, NC was the lncRNA GAS5 without biotin label. (**b**) The protein level of p21 and YBX1 after transfection with GAS5-E, GAS5-D or GAS5-B plasmids. (**c**) and (**d**) The cell cycle alteration after transfecting GAS5-E, GAS5-D or GAS5-B plasmids in HGC-27 (**c**) and in SGC-7901 (**d**). GAS5-E was the lncRNA GAS5 expression plasmid, GAS5-D was the lncRNA GAS5 mutant with its exon 12 deleted plasmid, and GAS5-B was the lncRNA GAS5 blank empty plasmid. *, p < 0.05.

A previous study demonstrated that the putative stem-loop region at 546–566 nt within exon 12 of lncRNA GAS5 was responsible for interacting with the GR protein, suggesting that exon 12 may be critical for lncRNA GAS5 to bind with proteins. Thus, we constructed a plasmid expressing GAS5 mutant without exon 12 named GAS5-D. The biotin-labeled lncRNA GAS5 mutant with exon 12 deletion named GAS5-DEL, which was transcribed from the GAS5-D plasmid for RNA pull-down experiment. As shown in Fig. 7a, lncRNA GAS5-DEL pulled down less YBX1 protein than full-length GAS5 (GAS5-FL), indicating that the GAS5-DEL mutation partly ablated the GAS5-YBX1 interaction. In addition, overexpressing GAS5 deleted mutant had a limited ability to enhance YBX1 and p21 protein levels (Fig. 7b). Compared with the full-length lncRNA GAS5, GAS5 deleted mutant failed to arrest the cell cycle at the G1 phase (Fig. 7c,d). These results indicate that the association between lncRNA GAS5 and YBX1 is necessary for lncRNA GAS5 to control YBX1 and p21 abundance and to regulate cell cycle, thus further establishing the critical roles of the lncRNA GAS5/YBX1/p21 pathway in stomach cancer development.

## Discussion

Recent studies have demonstrated that lncRNAs play important roles in the carcinogenesis and aggressive progression of human malignancies^12^,^25^. lncRNA GAS5 has gained increasing attention in cancer research because of its ubiquitously high expression during growth arrest^18^,^19^,^21^. The aberrant down-regulation of lncRNA GAS5 has been reported in breast cancer, renal cell carcinoma, bladder cancer, prostate cancer and pancreatic cancer^22^,^23^,^26^,^27^,^28^,^29^,^30^, however little is known regarding its expression in stomach cancer. Our results indicate that lncRNA GAS5 has lower expression in stomach cancer tissues than in their adjacent normal counterparts (Fig. 1c). Growth inhibition caused by starvation or rapamycin treatment elevated lncRNA GAS5 expression in HGC-27 and SGC-7901 stomach cancer cell lines (Fig. 1a,b). lncRNA GAS5 knockdown by siRNA abolished cell cycle arrest at the G1 phase (Fig. 2). Recently, Zhang *et al.* showed that lncRNA GAS5 expression is decreased in gastric cancer tissues and that lncRNA GAS5 down-regulation is associated with larger tumor size, advanced pathological stage and lower survival rate^13^. These findings further suggest that lncRNA GAS5 functions as a tumor suppressor and may represent a potential biomarker and new therapeutic target for stomach cancer. However, the mechanisms by which lncRNA GAS5 inhibits stomach carcinogenesis and development are largely unknown.

Previous studies have provided some clues regarding the mechanism by which lncRNA GAS5 confers tumor suppression. lncRNA GAS5 structurally mimics the GRE and acts as decoy to repress the expression of several anti-apoptosis genes induced by glucocorticoid^21^. Recently, lncRNA GAS5 was found to function as an endogenous sponge to attenuate oncogenic microRNA-21 expression in breast cancer^31^. In bladder cancer, lncRNA GAS5 associates with CDK6 and reduces both CDK6 mRNA and protein levels, resulting in the inhibition of cell proliferation^23^. To explore whether unknown mechanisms are responsible for the ability of lncRNA GAS5 to regulate cell cycle in stomach cancer, we performed an RNA pull-down assay and selected specific protein bands from the GAS5 complex for MS analysis (see Supplementary Figure S1 online). Among these MS-identified proteins, YBX1 had the highest score (see Supplementary Table S2 online). Then, Western blot analysis was conducted and confirmed that YBX1 was indeed present in the GAS5 complex. Furthermore RIP was also performed to confirm the reciprocal interaction between GAS5 and YBX1 (Fig. 3a–c). In addition, GR was also found in the GAS5 complex, and GAS5-DEL partly attenuated their association (see Supplementary Figure S3 online). Moreover, a weak p53 signal was detected in the GAS5 pull-down complex (see Supplementary Figure S2a online), and we found that p53 could interact with YBX1 by immunoprecipitating the cell lysate with p53 antibody (see Supplementary Figure S2b online). Recently, YBX1 has been reported to bind with GR^32^. According to these findings, we propose that complicated interactions may exist among GAS5, GR, YBX1, and p53. These proteins may cooperate together to exert functions.

In contrast to the previous observation that lncRNA GAS5 decreases CDK6 mRNA and protein levels in bladder cancer, our results indicate that lncRNA GAS5 knockdown reduced the YBX1 protein level (Fig. 3d) without affecting its transcription in stomach cancer (Fig. 3e). These results suggest that lncRNA GAS5 may interact with different proteins and use different mechanisms to regulate the cell cycle in specific tissues and cell types. Moreover, we found that depleting lncRNA GAS5 could accelerated YBX1 protein turnover in both HGC-27 and SGC-7901 cells (Fig. 3f,g), which enrich our knowledge on the versatile mechanisms of lncRNAs.

YBX1 is a multifunctional protein that regulates gene transcription and translation^33^,^34^. To assess whether YBX1 has any effect on the cell cycle in stomach cancer, YBX1 expression was knocked down with siRNAs in HGC-27 and SGC-7901 cells (Fig. 4a,c). We found that the percentage of cells that arrested at the G1 phase obviously decreased with YBX1 depletion (Fig. 4d,e). This result suggests that YBX1 participates in lncRNA GAS5-mediated cell cycle arrest.

The sequential activation of Cyclin/CDK complexes drives cell cycle progression in mammalian cells^35^,^36^. p21, one of the Cip/Kip family members, is well known for its critical role in negatively regulating the activity of Cyclin E/CDK2 complexes at the G1/S checkpoint and is activated by p53 and SP1^35^,^36^,^37^. Our results indicate that lncRNA GAS5-bound YBX1 is involved in transactivating p21. After YBX1 was down-regulated, both p21 mRNA and protein levels significantly decreased (Fig. 4b,c), which is similar to Okamoto *et al.’*s finding in prostate cancer^24^. Furthermore, we found that lncRNA GAS5 acted as the upstream effector that regulated YBX1 protein abundance. Consistent with the YBX1 down-regulation results, lncRNA GAS5 deletion also decreased p21 mRNA and protein levels (Fig. 5a-b). Moreover, the down-regulation of lncRNA GAS5 and of p21 in stomach cancer specimens positively correlated (Fig. 5c-d). Taken together, these findings suggest that lncRNA GAS5 expression is down-regulated during stomach carcinogenesis, which accelerates YBX1 protein turnover. Subsequently, the lower YBX1 protein levels decrease p21 expression and abolish G1 phase arrest.

To verify our findings, an lncRNA GAS5 plasmid and YBX1 siRNAs were co-transfected into the two cell lines. We found that YBX1 knockdown suppressed lncRNA GAS5-induced p21 elevation (Fig. 6a,c) and decreased the percentage of G1 phase arrest (Fig. 6d,e). These results confirm that YBX1 is essential for lncRNA GAS5 to enhance p21 expression and to trigger cell cycle arrest. Next, an lncRNA GAS5 mutant with exon 12 deleted was constructed to confirm our findings further. This GAS5-DEL mutant partly ablated the formation of the GAS5-YBX1 complex (Fig. 7a) and had a limited ability to enhance YBX1 and p21 protein abundance (Fig. 7b). Compared with the full-length lncRNA GAS5, overexpression of GAS5 mutant failed to arrest the cell cycle at the G1 phase (Fig. 7c,d). These results demonstrate that the interaction between lncRNA GAS5 and YBX1 is required to control YBX1 and p21 expression and to regulate the cell cycle. Thus, the lncRNA GAS5/YBX1/p21 pathway is important for controlling cell proliferation in stomach cancer.

In summary, we found that lncRNA GAS5 acts as a tumor suppressor that is down-regulated in stomach cancer. lncRNA GAS5 can bind to the YBX1 protein and regulate YBX1 protein turnover. The down-regulation of lncRNA GAS5 reduces the YBX1 protein level, which subsequently decreases YBX1-transactivated p21 expression and abolishes G1 phase arrest. Our findings provide new insight into the function and mechanism of lncRNA GAS5 in stomach cancer.

## Methods

### Clinical tissue specimens from stomach cancer patients

All paired tissue specimens were collected from 55 stomach cancer patients from November 2012 to March 2014 in Fudan University affiliated Huashan Hospital. Two pathologists confirmed the pathological diagnosis. All specimens were collected within 30 min after stomach resection and stored in liquid nitrogen immediately until RNA extraction. All 55 cancer tissues were identified as adenocarcinoma. Totally, there were 35 male patients and 20 female patients, with the age of 33 to 81, and on an average of 59. One was classified as stage 0, 5 as stage I, 18 as stage II, 29 as stage III and 2 as stage IV in accordance with cTNM Cancer Staging Manual. According to their differentiation status, 1 was well differentiated, 8 were moderate and 46 were poor (Supplementary Table S3 online). All patients enrolled in this study were informed and consent was given. The Human Research Ethics Committee of Huashan Hospital granted the ethics approval for this study. All the methods in the present study were in accordance with the approved guidelines and all the experimental protocols were approved by the Human Research Ethics Committee of Huashan Hospital.

### Cell cultures

The human stomach cancer cell lines (HGC-27 and SGC-7901) were purchased from Chinese Academy of Sciences (Shanghai, China). Both were cultured in ATCC-recommended 1640 cell culture medium (Invitrogen, 22400089). Cell culture was supplemented with 10% fetal bovine serum (FBS) (Invitrogen, 10099141), 50 U/mL Penicillin and 50 μg/mL Streptomycin (Invitrogen, 15070063). The cells were incubated at 37 °C in a humid atmosphere with 5% CO_2_.

### Antibodies, plasmids and siRNAs

For Western Blot, anti-YBX1 and anti-β-actin primary antibodies were purchased from Abcam (ab76149, ab52614), anti-p21 primary antibody and HRP-linked goat anti-rabbit IgG secondary antibody were purchased from Cell Signaling Technology (2947, 7074). For RIP assay, anti-YBX1 primary antibody was purchased from Cell Signaling Technology (9744), the normal mouse IgG was purchased from Merck Millipore (17-700).

A fragment containing the full length or exon 12 deleted lncRNA GAS5 sequence was constructed into the pcDNA3.1plasmid and was termed lncRNA GAS5 expression plasmid (GAS5-E) or lncRNA GAS5 exon 12 deleted plasmid (GAS5-D). The empty pcDNA3.1plasmid was used for negative control during plasmid transfection and was termed lncRNA GAS5 blank plasmid (GAS5-B). siRNAs against lncRNA GAS5 or YBX1(Supplementary Table S4 online) were synthesized by Shanghai GenePharma Co.

### Transfection

For siRNAs transfection, cells were seeded into 6-well plates at 10^6^ cells per well. 24 h later, cells were transfected with siRNAs or the negative control sequences using Lipofectaminev®2000 Transfection Reagent (Invitrogen, 11668027) at a final concentration of 50 nM according to the provided protocol. The medium was replaced 6 h after the transfection. For YBX1 siRNAs and lncRNA GAS5 plasmid cotransfection, cells were seeded into 6-well plates at 10^6^ cells per well. 24 h later, cells were cotransfected with lncRNA GAS5 plasmid and YBX1 siRNAs as indicatied. lncRNA GAS5 plasmid (GAS5-E or GAS5-B) amount was 200 ng per well. The final concentration of siRNAs for both cell lines was 50 nM. The medium was replaced 6 h after the transfection.

### RNA extraction, reverse transcription and quantitative real time-polymerase chain reaction (qRT-PCR)

Total RNA was extracted using the TRizol reagent (Invitrogen, 15596026) according to the provided protocol. RNA quantity and quality were determined by NanoDrop 2000c (Thermo Scientific, Waltham, MA, USA). Reverse transcription was performed using the PrimeScript™ RT reagent kit (TaKaRa, RR037A). The cDNA template was amplified by qRT-PCR using the SYBR Premix EX Taq™ II kit (TaKaRa, RR820A). The cycling condition was as follows, 95 °C for 60 s followed by 40 cycles of 94 °C for 10 s, 59 °C for 30 s and 72 °C for 30 s.

Glyceralde-hyde-3-phosphate-dehydrogenase (GAPDH) mRNA was used as an internal control. The primer sequences are presented in Supplementary Table S5 online.

### Cell cycle analysis

48 h after transfection, cells were trypsinized, harvested, and washed in cold PBS once and fixed in 75% ethanol at 4 °C for 2 h, then centrifuged, washed in cold PBS once and stained in BD Pharmingen™ PI/RNase staining buffer (BD Biosciences, 550825) at room temperature for 30 min in the dark, finally analyzed using a BD FACSCanto^TM^ Flow Cytometer (BD Biosciences, San Jose, CA, USA). For each sample, 10^5^ events were counted.

### Western blot

Briefly, cells were harvested and lysated in RIPA buffer containing protease inhibitor phenylmethanesulfonyl fluoride (PMSF) (Beyotime, P0013B). The concentration was determined using Enhanced BCA Protein Assay Kit (Beyotime, P0010). Protein was denatured at 100 °C for 10 min, electrophoretically separated (50 μg per lane) on 12% SDS–PAGE (Sangon Biotech, SD6013) and then transferred onto a nitrocellulose Western blot membrane. The membrane was blocked in 5% skim milk at room temperature for 30 min, incubated with a monoclonal primary antibody at 4 °C overnight. After being washed three times in 15 min, the membrane was incubated with a secondary antibody for 1 h at room temperature, washed three times in 15 min, and incubated with Immobilon^TM^ Western chemiluminescent HRP substrate (Merck Millipore, WBKLS0100) for 2 min at room temperature. Finally the membrane was exposed to Kodak film in dark room to evaluate the protein expression. The bands of Western blot were scanned to quantify their density with the software Quantity One (Version 4.4.1).

### Protein turnover analysis

HGC-27 and SGC-7901 cells were transfected with lncRNA GAS5 siRNA oligos, 24 h after transfection, cycloheximide(CHX) was added to the cell culture at a final concentration of 12.5 μg/mL. After treatment with CHX, the cells were harvested at the indicated interval as shown in Fig. 3f,g. Western blot was performed to detect YBX1 protein levels.

### RNA pull-down assay

The RNA sequences used for RNA pull-down assay were transcribed from their corresponding plasmids in vitro, and biotin labeled using the Biotin RNA Labeling Mix (Roche, 11685597910) and T7/SP6 RNA polymerase (Roche, 10881767001, 10810274001), treated with RNasefree DNase I (Roche, 04716728001) and purified with RNeasy Plus Mini Kit (Qiagen, 74134). Protein (1 mg) from HGC-27 cell extracts was mixed with 3 μg of biotin-labeled RNA, incubated with Dynabeads® M-280 Streptavidin (Invitrogen, 11205D) at 4 °C for 3 h. Finally, the retrieved proteins were resolved by SDS-PAGE and silver stained. The specific bands were excised and analyzed by Mass Spectrometry (MS).

### RIP assay

RIP assay was performed using a Magna RIP™ RNA Binding Protein Immunoprecipitation Kit (Merck Millipore, 17-700) according to the provided protocol. (The electronic version of this protocol could be downloaded from this website link http://www.merckmillipore.com/CN/en/product/Magna-RIP%E2%84%A2-RNA-Binding-Protein-Immunoprecipitation-Kit,MM_NF-17-700#anchor_BRO). Briefly, the YBX1-protein-precipitated RNAs were reverse transcribed using the PrimeScript™ RT reagent kit (TaKaRa, RR037A). The cDNA templates were amplified by qRT-PCR using the SYBR Premix EX Taq™ II kit (TaKaRa, RR820A). The thermal condition was shown above. The cDNA templates were also amplified by Polymerase Chain Reaction (PCR) using 2×EasyTaq® PCR SuperMix (TransGen Biotech, AS111-01). The thermal condition was as follows, 94 °C for 3 min, after 20 cycles of 94 °C for 20 s, 60 °C for 30 s and 72 °C for 30 s, then the mixture was heated to 72 °C and held for 10 min, then 4 °C for 10 min. The products were resolved on 3% agarose gel electrophoresis.

### Statistic analysis

All data were analyzed using SAS 8.0. In stomach cancer specimens, the significance of lncRNA GAS5 and p21 mRNA expression was analyzed using Wilcoxon signed rank sum test, the correlation between lncRNA GAS5 and p21 mRNA was analyzed using Spearman rank correlation analysis. The significance of experiments was analyzed using Student’s t-test. p < 0.05 was considered statistically significant. The data were acquired from at least three independent experiments.

## Author Contributions

Y.C.L. and J.Z. carried out all the experimental studies, drafted the manuscript and performed the statistical analysis. Y.C.L., W.H.Z., C.E.H. and J.G. collected all specimens. G.J.H. and Y.Z. conceived the study, participated in its design and coordination and revised the manuscript. All authors read and approved the final manuscript.

## Additional Information

**How to cite this article**: Liu, Y. *et al*. IncRNA GAS5 enhances G1 cell cycle arrest via binding to YBX1 to regulate p21 expression in stomach cancer. *Sci. Rep.* **5**, 10159; doi: 10.1038/srep10159 (2015).

## Supplementary Material

Supplementary Information
