# Supplementary material for: lncRNA GAS5 enhances G1 cell cycle arrest via binding to YBX1 to regulate p21 expression in stomach cancer
Source: Sci Rep. 2015 May 11;5:10159. doi: 10.1038/srep10159 (PMC4426700; doi:10.1038/srep10159)
Supplement: Supplementary Information [file srep10159-s1.doc]

# lncRNA GAS5 enhances G1 cell cycle arrest via binding to YBX1 to regulate p21 expression in stomach cancer

Running title,

lncRNA GAS5/YBX1/p21 pathway in stomach cancer

Yongchao Liu^1#^, Jing Zhao^2#^, Wenhong Zhang^2^, Jun Gan^1^, Chengen Hu^1^, Guangjian Huang^1*^, Ying Zhang^2,3*^

1, Department of General Surgery, Huashan Hospital, Fudan University, Shanghai, China

2, Department of Infectious Diseases, Huashan Hospital, Fudan University, Shanghai, China

3, Department of Molecular Microbiology and Immunology, Bloomberg School of Public Health, Johns Hopkins University, Baltimore, Maryland, USA

**#These authors contribute equally to this work**

***Correspondence to:**

**Guangjian Huang:**

Department of General Surgery, Huashan Hospital, Fudan University

**Email address:** guangjian_huang@hotmail.com

**Ying Zhang:**

Department of Molecular Microbiology and Immunology, Bloomberg School of Public Health, Johns Hopkins University

**Email address:** yzhang@jhsph.edu

**Supplementary information:**

Table S1. Significance of deregulated lncRNA GAS5 and p21 mRNA.

| **Factors** | **No of down regulated** | **No of up regulated** | **p value** |
| --- | --- | --- | --- |
| **lncRNA GAS5** | 41 (74.5%) | 14 (25.5%) | 0.0002 |
| **p21** | 40 (72.7%) | 15 (27.3%) | 0.0055 |

In each paired specimen (cancer and normal tissues are from the same patient), the expression level of cancer compared with its normal tissue more than 1 was considered to be up-regulated, or it was considered to be down-regulated. p<0.05 was considered statistically significant.

Table S2. Mass spectrometry analysis of the proteins pulled down by lncRNA GAS5.

| **Number** | **Accession** | **Mass** | **Score** | **Description** |
| --- | --- | --- | --- | --- |
| 1. | gi|34098946 | 35903 | 82 | nuclease-sensitive element-binding protein 1 [Homo sapiens] |
| 2. | gi|54040031 | 35903 | 82 | RecName: Full=Nuclease-sensitive element-binding protein 1; AltName: Full=CCAAT-binding transcripti |
| 3. | gi|119627554 | 35903 | 82 | Y box binding protein 1, isoform CRA_b [Homo sapiens] |
| 4. | gi|76779237 | 35903 | 82 | Y box binding protein 1 [Homo sapiens] |
| 5. | gi|68534659 | 35903 | 82 | Y box binding protein 1 [Homo sapiens] |
| 6. | gi|58477789 | 35903 | 82 | Y box binding protein 1 [Homo sapiens] |
| 7. | gi|47940506 | 35903 | 82 | Y box binding protein 1 [Homo sapiens] |
| 8. | gi|47125298 | 35903 | 82 | Y box binding protein 1 [Homo sapiens] |
| 9. | gi|41350935 | 35903 | 82 | Y box binding protein 1 [Homo sapiens] |
| 10. | gi|23468206 | 35903 | 82 | Y box binding protein 1 [Homo sapiens] |
| 11. | gi|15929560 | 35903 | 82 | Y box binding protein 1 [Homo sapiens] |
| 12. | gi|14714588 | 35903 | 82 | Y box binding protein 1 [Homo sapiens] |
| 13. | gi|12803207 | 35903 | 82 | Y box binding protein 1 [Homo sapiens] |
| 14. | gi|454152 | 35903 | 82 | similar to dbpB protein [Homo sapiens] |
| 15. | gi|340419 | 35391 | 82 | Y box binding protein-1 [Homo sapiens] |
| 16. | gi|23343289 | 35391 | 82 | unnamed protein product [Homo sapiens] |
| 17. | gi|21522730 | 35391 | 82 | unnamed protein product [Homo sapiens] |
| 18. | gi|181486 | 39954 | 81 | DNA-binding protein B, partial [Homo sapiens] |
| 19. | gi|47939143 | 29357 | 65 | YBX1 protein, partial [Homo sapiens] |
| 20. | gi|33875177 | 29357 | 65 | YBX1 protein, partial [Homo sapiens] |

Protein score greater than 71 is significant (p<0.05).

Table S3. The expression levels of lncRNA GAS5 and p21 mRNA in terms of clinical and pathological characteristics.

| **Item** | **No of cases** | **lncRNA GAS5** | | ***p21*** | |
| --- | --- | --- | --- | --- | --- |
|  |  | ↑ | ↓ | ↑ | ↓ |
| **Gender** | | | | | |
| **Male** | 35 | 7 | 28 | 8 | 27 |
| **Female** | 20 | 7 | 13 | 7 | 13 |
| **Age (years)** |  | | | | |
| **≤65** | 38 | 10 | 28 | 11 | 27 |
| **＞65**  **Diameter(cm)** | 17 | 4 | 13 | 4 | 13 |
| **≤4** | 33 | 7 | 26 | 10 | 23 |
| **＞4**  **cTNM** | 22 | 7 | 15 | 5 | 17 |
| **0** | 1 | 0 | 1 | 0 | 1 |
| **Ⅰ** | 5 | 2 | 3 | 2 | 3 |
| **Ⅱ** | 18 | 5 | 13 | 6 | 12 |
| **Ⅲ** | 29 | 6 | 23 | 7 | 22 |
| **Ⅳ** | 2 | 1 | 1 | 0 | 2 |
| **Differentiation** | | | | | |
| **Well** | 1 | 0 | 1 | 0 | 1 |
| **Moderate** | 8 | 2 | 6 | 4 | 4 |
| **Poor** | 46 | 12 | 34 | 11 | 35 |

In each paired specimen (cancer and normal tissues are from the same patient), the expression level of cancer comparing with its normal tissue more than 1 was considered to be up-regulated and represented with ↑, or it was considered to be down-regulated and represented by ↓.

Table S4. siRNAs sequences.

| **Item** | **Sense(5’-3’)** | **Antisense(5’-3’)** |
| --- | --- | --- |
| **lncRNA GAS5 1#** | GGACCAGCUUAAUGGUUCUTT | AGAACCAUUAAGCUGGUCCTT |
| **lncRNA GAS5 2#** | GCAGACCUGUUAUCCUAAATT | UUUAGGAUAACAGGUCUGCTT |
| **YBX1 1#** | CUGCCAUAAAGAAGAAUAATT | UUAUUCUUCUUUAUGGCAGTT |
| **YBX1 2#** | GCAGACCGUAACCAUUAUATT | UAUAAUGGUUACGGUCUGCTT |
| **YBX1 3#** | CGGCAAUGAAGAAGAUAAATT | UUUAUCUUCUUCAUUGCCGTT |
| **Negative control** | UUCUCCGAACGUGUCACGUTT | ACGUGACACGUUCGGAGAATT |

Table S5. Primer sets for qRT-PCR or PCR.

| **Gene** | **Primer** | **Sequence(5’-3’)** |
| --- | --- | --- |
| **lncRNA GAS5** | forward | | CCATGGATGACTTGCTTGGG | | --- | |
|  | reverse | \| TGCATGCTTGCTTGTTGTGG \| \| --- \| |
| **p21** | forward | | TGTCCGTCAGAACCCATG | | --- | |
|  | reverse | \| TGGGAAGGTAGAGCTTGG \| \| --- \| |
| **YBX1** | forward | GGACAAGAAGGTCATCGCAAC |
|  | reverse | \| TCTCCATCTCCTACACTGCGA \| \| --- \| |
| **GAPDH** | forward | | TCGACAGTCAGCCGCATCTTCTTT | | --- | |
|  | reverse | \| ACCAAATCCGTTGACTCCGACCTT \| \| --- \| |

**
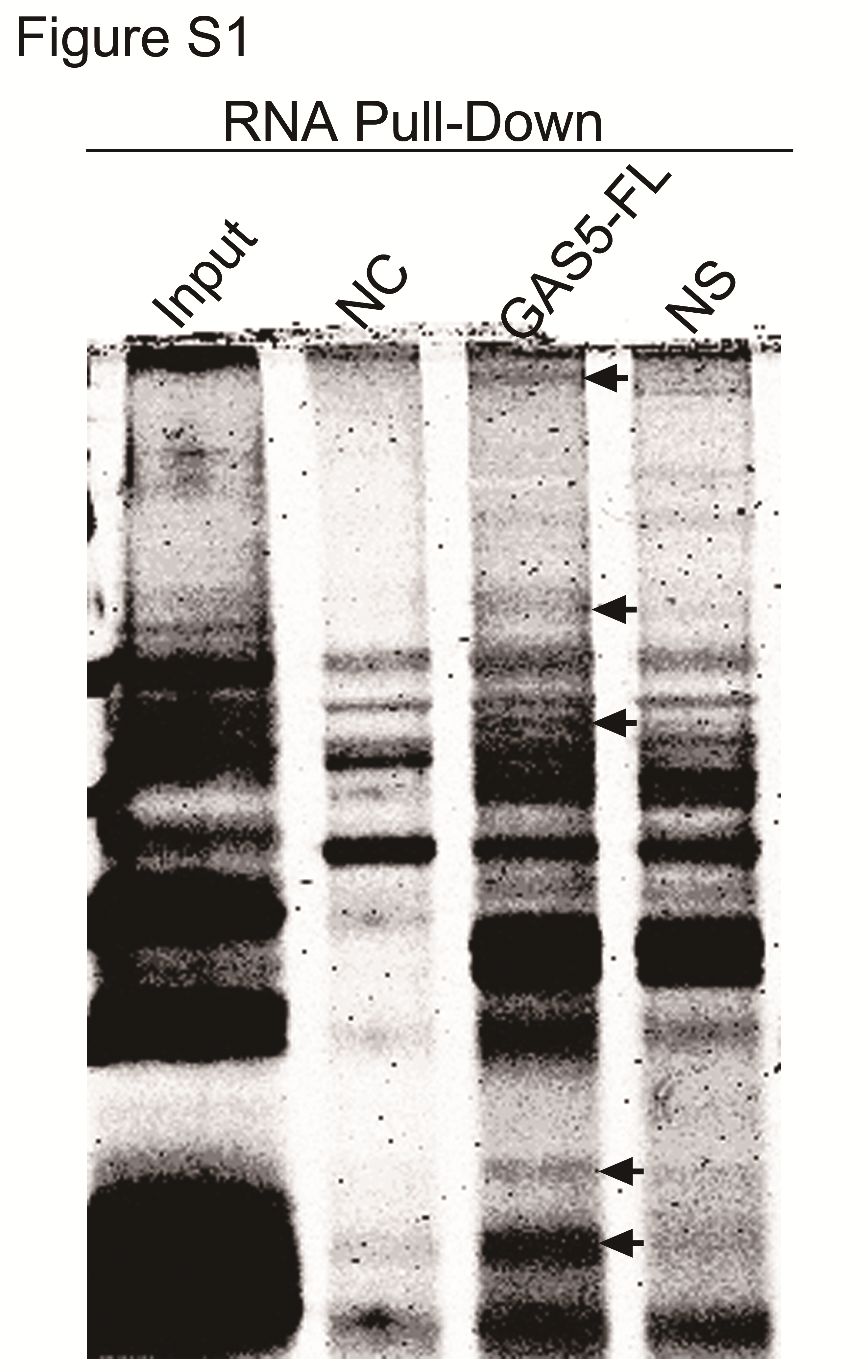
**

**Figure S1. The silver-stained gel of RNA pull-down assay**

The protein pulled down by GAS5-FL (the biotin-labeled lncRNA GAS5), NS (the biotin labeled non-sense RNA with similar length to GAS5) and NC (the lncRNA GAS5 without biotin labeled). Compared with NS and NC groups, five distinct protein bands in GAS5-FL were cut out for Mass Spectrometry (MS) analysis as the arrow indicated.


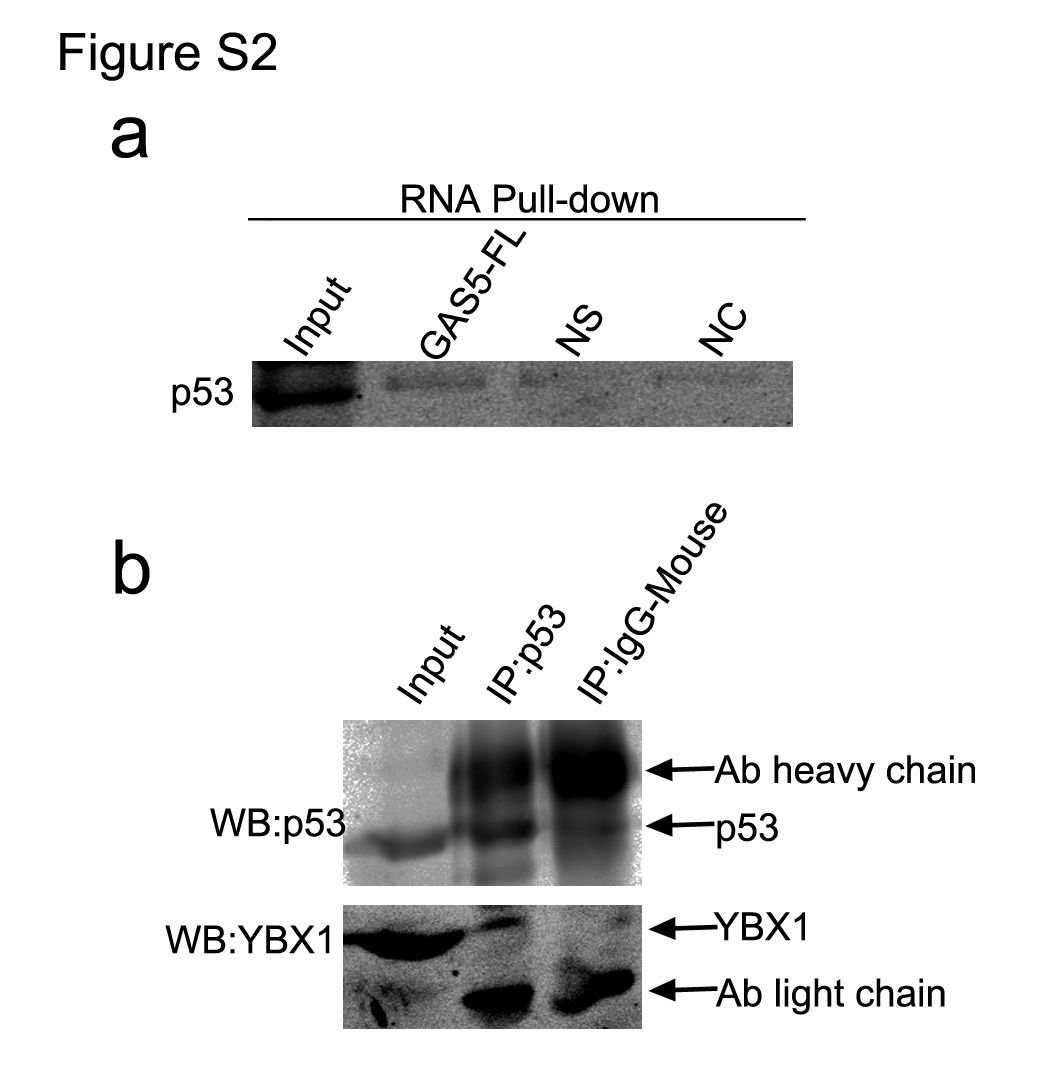


**Figure S2. lncRNA GAS5 RNA pull-down complex with p53 and co-immunoprecipitation between p53 and YBX1.**

(a) Western Blot detected p53 from the lncRNA GAS5 pull-down complex. (b) YBX1 could be co-immunoprecipitated by p53 primary antibody.


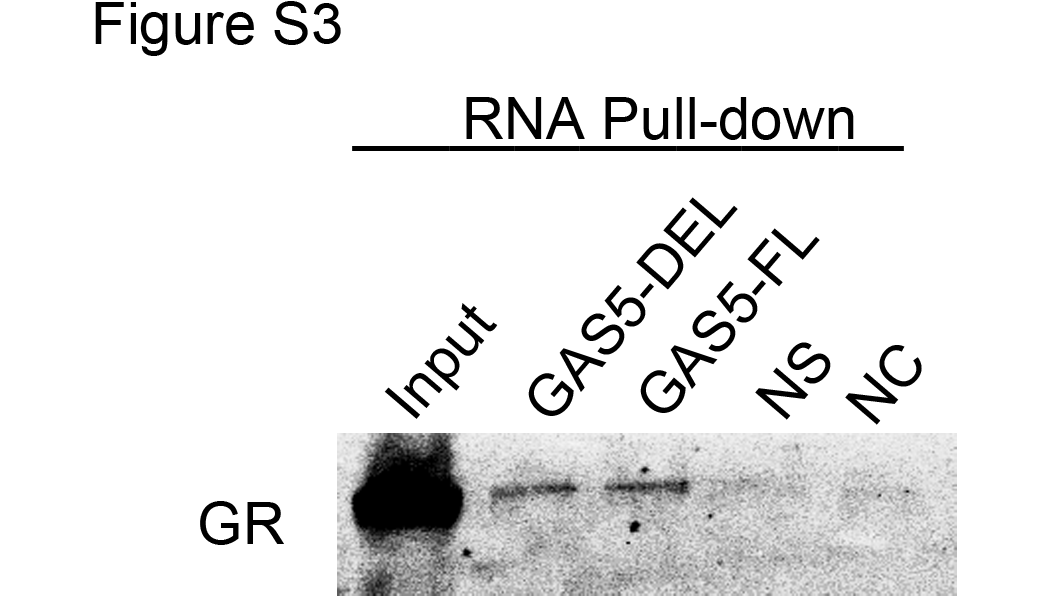


**Figure S3. Western Blot with GR antibody detected GAS5-FL and GAS5-DEL RNA pull-down complex.**

GAS5-DEL was the biotin labeled lncRNA GAS5 mutant with its exon 12 deletion, GAS5-FL the was biotin labeled full length lncRNA GAS5, NS was the biotin labeled non-sense RNA with similar length to GAS5, NC was the lncRNA GAS5 without biotin label.
